# Urinary Exosomes and Their Cargo: Potential Biomarkers for Mineralocorticoid Arterial Hypertension?

**DOI:** 10.3389/fendo.2017.00230

**Published:** 2017-09-08

**Authors:** Eric R. Barros, Cristian A. Carvajal

**Affiliations:** ^1^Center of Translational Endocrinology (CETREN), Faculty of Medicine, Endocrinology Department, Pontificia Universidad Católica de Chile, Santiago, Chile

**Keywords:** arterial hypertension, exosomes, biomarker, water-electrolyte balance, microRNA, urine, sodium channels

## Abstract

Arterial hypertension (AHT) currently affects approximately 40% of adults worldwide, and its pathological mechanisms are mainly related to renal, vascular, and endocrine systems. Steroid hormones as aldosterone and cortisol are highly relevant to human endocrine physiology, and also to endocrine hypertension. Pathophysiological conditions, such as primary aldosteronism, affect approximately 10% of patients diagnosed with AHT and are secondary to a high production of aldosterone, increasing the risk also for cardiovascular damage and heart diseases. Excess of aldosterone or cortisol increases the activity of the mineralocorticoid receptor (MR) in epithelial and non-epithelial cells. Current research in this field highlights the potential regulatory mechanisms of the MR pathway, including pre-receptor regulation of the MR (action of 11BHSD2), MR activating proteins, and the downstream genes/proteins sensitive to MR (e.g., epithelial sodium channel, NCC, NKCC2). Mineralocorticoid AHT is present in 15–20% of hypertensive subjects, but the mechanisms associated to this condition have been poorly described, due mainly to the absence of reliable biomarkers. In this way, steroids, peptides, and lately urinary exosomes are thought to be potential reporters of biological processes. This review highlight exosomes and their cargo as potential biomarkers of metabolic changes associated to mineralocorticoid AHT. Recent reports have shown the presence of RNA, microRNAs, and proteins in urinary exosomes, which could be used as biomarkers in physiological and pathophysiological conditions. However, more studies are needed in order to benefit from exosomes and the exosomal cargo as a diagnostic tool in mineralocorticoid AHT.

## Arterial Hypertension (AHT)

Arterial hypertension is a multifactorial disease with a complex pathogenesis involving several systems. Different etiologies of AHT are known to occur from the interplay between genetic and environmental factors that lead to changes in biological pathways and eventually trigger this complex disorder that primarily involves the cardiovascular system (1–3). AHT is a major risk factor for stroke, myocardial infarction, heart failure, and end-stage renal disease. Worldwide, approximately 40% of adults over 25 years old are affected by AHT, contributing to 45–50% of deaths due to heart disease and stroke (4, 5), making AHT a major concern for public health, particularly in western countries (6–11). The pathogenesis of AHT involves the renal, vascular, and endocrine systems (12–15), affecting mainly sodium-water reabsorption and arterial vasoconstriction. Pathological conditions, such as primary aldosteronism (PA), are responsible for up to 5–10% of patients diagnosed with AHT, and involve an increased production of aldosterone that leads to AHT, cardiovascular damage, heart diseases (16–18), and renal and immune alterations (19–23). Aldosterone is a mineralocorticoid hormone with non-genomic and genomic actions; the latter through the mineralocorticoid receptors (MR) can alter sodium transport in renal collecting ducts, increase water uptake, blood volume, and eventually raise blood pressure. Along with effects of aldosterone in AHT, different studies in cells, animal models, and human trials through the analysis of serum and urinary markers have confirmed the pathogenic role of aldosterone on inflammation, endothelial dysfunction, oxidative stress, and fibrosis (20, 24, 25). Other factors, as novel proteins associated to aldosterone have also been reported to independent activate or enhance the MR action, such as a small GTP-ase Rac1 (26, 27), GPER (28), and the co-activator RACK1 (29). However, only Rac1 has been described as component of exosomes (http://exocarta.org/download). GPER (previously known as GPR30) is a recently recognized G protein-coupled receptor implicated in mediating some of the rapid effects of steroid hormones, especially aldosterone. GPER protein is activated by aldosterone, but its relation with exosomes and mineralocorticoid AHT has not been studied to date.

Most studies about primary AHT have being focused in genetic alterations associated with the onset and progression of AHT affecting cardiac, endocrine, and renal systems (1, 2, 30–32). Gene-specific (31, 33, 34), genome-wide association (35–39), and epigenetic studies (40, 41) support the knowledge about the genetic components related to AHT. Several phenomena that regulate gene expression through genetics and epigenetics are emerging to understanding of AHT development (40, 42, 43). Most of the studies addressing the role of epigenetics in human AHT (44, 45) have focused their interest on DNA methylation (46, 47) and non-coding RNAs such as microRNAs (miRNAs) (41).

Gene expression is a coordinated system regulating specific synthesis and interaction of RNA, miRNAs, and proteins. All of them can be also carried and potentially transferred to other cells (recipient cells) through nanovesicles called exosomes, where they regulate further cellular and metabolic processes (48). Identification of cell-specific RNA and proteins contained in exosomes isolated from different biofluids, may be a promising biological tool to identify early signs of AHT (49–51). This review highlight exosomes and their cargo as potential biomarkers or biological reporters of metabolic changes associated to mineralocorticoid AHT.

## Exosomes are Carriers of Biological Information

Exosomes and microvesicles are involved in several metabolic processes (52) initially proposed in the 1980s (53), described in tissues (54), body fluids (55) and considered to be vehicles for eradicating cell waste products (56). They are currently defined as extracellular vesicles of endosomal origin, with a spherical shape and a phospholipid bilayered structure of 30–150 nm diameter, carrying exosomal markers (e.g., CD63, HSP70) and a buoyant density of 1.23–1.16 g/L (57–59). Exosomes have important functions in immunology, cancer, coagulation, and many others aspects of human physiology, as carriers of information including, proteins (60), lipids (61), mRNA, miRNA (48), and DNA (62).

Exosomes act through receptor–ligand interactions, by attaching/fusing with the target-cell membrane or by being internalized by the recipient cells (63) performing cell-to-cell communication and the intercellular exchange of proteins and nucleic acids, with relative stability against proteinases and RNAses (48). mRNAs horizontally transferred from exosomes to neighboring cells can be translated into proteins, and miRNAs can regulate acceptor mRNA expression (64). Exosomes contain a specific subset of cellular proteins, some of which depend on the cell type of origin, and others that are only found in exosomes regardless of the cell type of origin (57).

## Exosomes and Exosomal Cargo are Potential Novel Biomarkers for Arterial Hypertension

Arterial hypertension is mainly associated with alterations in the cardiovascular and renal systems, in which there is great interest for discovering new biomarkers, highlighting the potential role of exosomes. Human blood, saliva, and urine are biofluids that constitute a source of non-invasive, convenient and easy to access biomarkers that can be collected many times over long periods of time. Spot urine and 24-h urine is the focus for the identification of novel peptide, steroidal, or exosomal biomarkers with a potential role in diagnosis and classification of diseases related to renal system (65, 66).

Recent urinary proteomic studies have identified potential protein biomarkers of renal disease (67) such as nephrin (68) or podocin (69), but none of them have been translated into regular clinical practice. This is probably because free urinary proteins are often scarce, and frequently reabsorbed in the tubular renal systems or subjected to proteolytic digestion (70), similar to urinary RNA, which is degraded by RNAses in renal tissues (71); therefore, exosomes and their cargo, which are protected by a plasmatic membrane that is resistant to these influences, seem to be a suitable source of urinary biomarkers (72).

## Urinary Exosomes, Renin–Angiotensin–Aldosterone System (RAAS) and Mineralocorticoid AHT

Urinary exosomes originate from cells lining the nephron lumen and the urinary tract (70). Plasmatic exosomes cannot cross the glomerular filtration apparatus; therefore, urinary exosomes originate exclusively from luminal epithelial renal cells (73). Proteins detected in urinary exosomes are a reflection of the proteins in renal tissues (74, 75) and from acute injured sites distant from the urinary tract (e.g., liver injury). The former increase their protein abundance upon stimulation of the renin-angiotensin-aldosterone system (RAAS), which is an important blood pressure regulator (65). Exosomal RNAs, miRNAs, and proteins can mirror gene expression changes in kidney diseases (70, 76) promising to be effective and non-invasive biomarkers for renal disease and may be used as surrogate markers of RAAS activation, affecting expression of the epithelial sodium channel (ENaC) (OMIM: 600228), the thiazide-sensitive sodium-chloride-cotransporter (NCC) (OMIM: 600968) (77, 78) among others. The complexity of the urinary proteome hinders the detection of low-abundance proteins that may have pathophysiological relevance; therefore, the evaluation of urine exosomal proteins, which represent approximately 3% of the whole urine proteome, reduces the complexity of studying the whole urine proteome (79). These urinary proteins may originate from renal physiological processes that occur within the kidneys and/from exosome secretion (72). Comprehensive studies have been conducted on the proteome of urinary exosomes, revealing that they contain a variety of cell-specific proteins/transporters from the kidney and from the urogenital tract (80, 81), that could be useful in the diagnosis of mineralocorticoid AHT.

## Water-Electrolytic Balance: Taking Advantage of Exosomes

### Sodium Transporters

**Figure 1:**
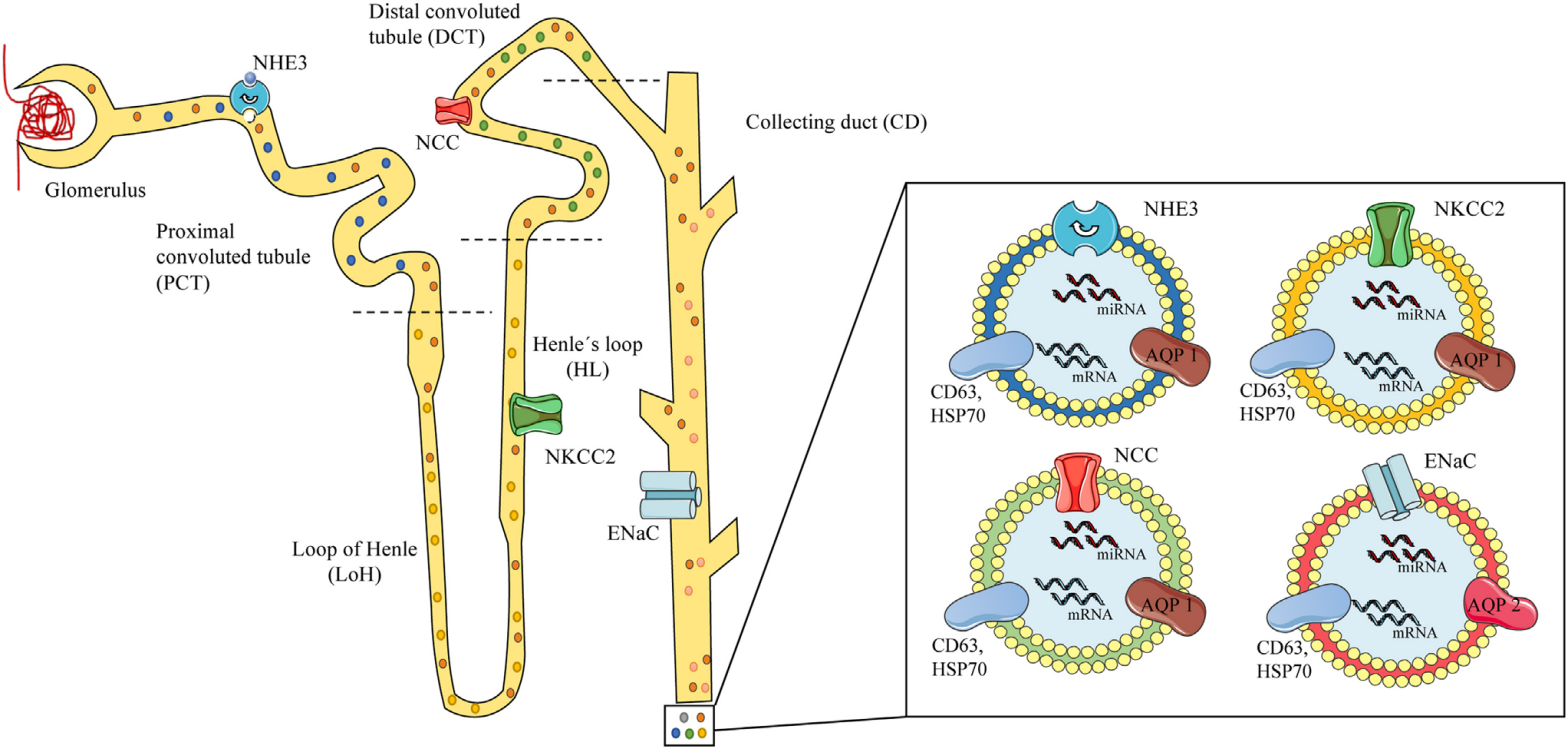
Scheme of urinary exosomes released from different nephron segments. On the left, a scheme of the glomerulus and nephron showing exosomes released and traveling through the tubules carrying proteins or RNAs from the different segments of the nephron, containing specific channels associated to mineralocorticoid arterial hypertension. Specific local expression of sodium channels may be associated to the exosomal cargo: NHE3 from the proximal convoluted tubule (PCT) (blue exosome), NKCC2 from the loop of Henle (LoH) (yellow exosome), NCC from the distal convoluted tubule (green exosome), and epithelial sodium channel from the collecting duct (red exosomes). On the right, urinary exosomes (bilayered nanovesicles of 30–120 nm diameter) contain specific proteins (e.g., sodium channels), mRNA, and microRNA (miRNA), and show typical markers (CD63, HSP70) along with renal proteins (AQP1–2). Model based on references (49–51, 73, 80).

There is considerably more research on the use of urinary exosomes than circulating exosomes for the diagnosis of hypertension, probably because most sodium transporter are present on the apical plasma membrane of the kidney epithelium and urinary exosomes are released to the lumen of the nephron under hormonal regulation. Sodium channels and specific miRNAs expression in exosomes are susceptible to the action of aldosterone alone and the RAAS (49, 65, 82). The RAAS and the kidneys play a pivotal role in blood pressure regulation (83) with sodium channels acting as crucial elements in the regulation of the electrolyte balance and blood pressure (84, 85). Some of the main players in sodium/water balance are the NHE3 (*SLC9A3*, sodium-hydrogen exchanger 3) (86) present in the renal proximal tubule, the Na–K–Cl cotransporter NKCC2 *(SLC12A1*) in the thick ascending loop of Henle (LoH) (84, 87, 88) and the NCC *(SLC12A3)* along with the ENaC (*SCNN1*) on the distal nephron (distal convoluted tubules and collecting duct). Altered function of these leads to hypertensive syndromes, such as Liddle (increased ENaC activity) (89) and Gordon (WNK4-NCC) (90, 91), or hypotensive syndromes, such as Gitelman (NCC) (92) and Bartter (NKCC2) (30) (see Figure 1).

Patients diagnosed with Gordon syndrome had a fourfold increase in the abundance of NCC in urinary exosomes when compared to controls (90); a recent publication in patients under exogenous mineralocorticoid (fludrocortisone) administration showed a reduction of 48% in the phosphorylated NCC (pNCC)/NCC ratio along with a rapid increase in the abundance of NCC and pNCC in urinary exosomes, possibly through the WNK pathway (77). Interestingly, Castagna et al. in 2015 showed that exosomal and urinary NCC is under circadian regulation (93). Urinary exosomes from patients diagnosed with the Gitelman and Bartter type-1 syndromes, showed almost undetectable levels of NCC and NKCC2 proteins, making feasible to discriminate between the syndromes and their severity through the exosomal protein content (94).

Urinary exosomes from mildly hypertensive patients on a low-sodium diet (activated RAAS) showed that a 11.4% of their total protein content changes (316 out of 2,775 proteins), with 4.1% of the proteins increasing and 7.3% decreasing the expression level. Here, the abundance of NCC, and the α, β, and γ subunits of ENaC increased under low-sodium diet or aldosterone infusion correlating with plasma aldosterone and urinary Na/K ratio (65). This communication also highlights the presence of the γENaC_[112–122]_ peptide that increases nearly 20-folds by both challenges (LS diet or under aldosterone infusion) and correlates with plasma aldosterone and urinary Na/K ratio, while αENaC and NCC from urinary exosomes did not change under the same stimuli (65). Further evidence linking ENaC, exosomes, and AHT comes from Nielsen et al. (95) who studied pregnant women in normal and preeclamptic conditions (95) and from Olivieri et al. (96) who measured urinary exosomal prostasin from healthy subjects and found a correlation with aldosterone to renin ratio and urinary sodium (96).

Rat models of sodium imbalance show a correlation between the renal-tissue expression of NCC and NKCC2, and the expression of the same proteins on urinary exosomes (75). Urinary exosomes from rats under aldosterone infusion or low-sodium diet increased the levels of pNCC (97), similar to urinary exosomes from patients with PA, who had more pNCC than hypertensive patients, suggesting a possible role for exosomes as a PA biomarker (93). Similarly, an animal model of Sprague-Dawley rats under sodium restriction showed increased expression of fully processed ENaC, with the α and γ subunits in fully cleaved states, and the β-ENaC fully glycosylated in urinary exosomes (98).

### Aquaporins

Aquaporins (AQPs) are renal membrane proteins involved in the transfer of water and solutes across cell membranes, influencing urine formation and water handling. At least eight isoforms are reported in renal tissues (AQP 1–4, 6–8, 11) (99). AQP1 is expressed in the kidney’s proximal tubule cells, the thin descending LoH and the descending vasa recta, while AQP2 (anti-diuretic hormone-regulated water channel) is expressed in the principal cells of connecting tubules and collecting ducts (100–102).

Urinary exosomes have been observed carrying AQP1 and AQP2 (80, 103). AQP2 is under circadian regulation decreasing in the morning and increasing in the afternoon/evening (93). The exosomal protein levels of AQP1–2 correlate with the renal expression and reflect their action on renal cells (104). The other AQPs have not been found in urinary extracellular vesicles (99). Decreased levels of AQP1 have been observed in urinary exosomes from a renal rat ischemia reperfusion injury model, from the urine of human patients after renal transplantation (105) and in cultured cells after exposure to acetazolamide (diuretic acting on the nephron’s proximal tubules) (106). In contrast, acetazolamide increases AQP1 in rat urinary exosomes without a decrease in the renal expression (107). Long-term effect of vasopressin or vasopressin analogs promotes extracellular vesicle uptake in renal epithelial cells (108), extracellular vesicle release in collecting duct cells, and enhance urinary excretion of exosomal AQP2 (109, 110) in murine kidney collecting duct cells (mCCDC11), rodents, and patients with central diabetes insipidus (111). The short-term effects of these hormones increase the abundance of urinary exosomal AQP2 protein (101) but reduces it under hypervolemic states (112). Regarding the mineralocorticoid pathway, there is a controversial relation between AQP2 expression, aldosterone and the MR activation (113).

### Renin–Angiotensin–Aldosterone System

As stated previously, the RAAS is a major regulator of blood pressure acting over the renal, vascular, cardiac, and adrenal systems. Angiotensin II (Ang II) and the Ang II type I receptor (AT1R) play key roles that could be being driven by exosomes. In 2015, Pironti et al. showed that either cardiac overload or Ang-II stimulation, induced the release of exosomes to the circulation (mainly from cardiomiocytes) carrying functional AT1R, that move to cardiac/skeletal myocytes and resistance vessels, further regulating blood pressure (114), and probably improving their sensitivity to RAAS (82).

Angiotensin II besides elevating blood pressure, is also associated with inflammation mediated end-organ damage and fibrosis in AHT; hypertensive rat models (Ang II or l-NAME infused) release serum exosomes with decreased levels of miRNA-17 (ICAM-1 negative regulator) that when cultured with human coronary artery endothelial cells increase the protein expression of ICAM-1 and PAI-1, which are essential pro-inflammatory factors in vascular inflammation (115) providing evidence that hypertensive-related endothelial damage may be due to exosomes and their cargo. The association between urinary exosomes and renal RAAS has been poorly studied and only indirect evidence has been reported in the literature (65, 80, 97).

## Urinary Exosomes as Carriers of miRNAs

MicroRNAs are endogenous small RNA molecules of approximately 22 nucleotides that can control a target gene transcriptionally and posttranscriptionally (116) by complementarily binding the 3′UTR of target mRNA (117, 118). miRNAs are involved in cellular processes including proliferation, development, metabolism, differentiation, and apoptosis. Individual miRNAs may regulate hundreds of genes, collectively 50–60% of the total transcriptome (119), suggesting that miRNAs can have pleiotropic biological effects. Deregulation of miRNA expression is linked to many human pathological conditions; however, few studies have evaluated the relationship between miRNA expression and regulation of the MR pathway, which has been associated mainly to gene expression downregulation at pre-receptor level, as occurs with 11BHSD2 (120, 121).

Different studies relate renal expression of miRNAs and AHT (122). In 2013, Gildea et al. studied the miRNAome of urinary exosomes (49) and found 45 miRNAs likely to be potential biomarkers that correlated with salt sensitivity or inverse salt sensitivity of blood pressure. Some of these miRNAs regulate signaling pathways associated to AHT, reflecting the metabolic activity of the kidney and particularly sodium handling (see Table 1).

**Table 1:** Studies reporting miRNAs associated to genes or signaling pathways related to AHT.

Cell type or zone	Gene	NCBI ID	OMIM	MicroRNA (miRNA) affecting gene	Sample source	Function related to AHT	Reference
Collecting duct tubule (CDT) cells	*NEDD4L*	NG_029954.1	606384	miRNA-30a-5p	Urine from healthy volunteers	Aldosterone regulated sodium reabsorption	(51)
	*HSD11B2*	NG_016549.1	218030	miRNA-4474-3p	Urine from healthy volunteers	Mineralocorticoid receptor (MR) activation	(51)
	*SCNN1A*	NG_011945.1	600228	miRNA-4747-5p	Urine from healthy volunteers	αENaC-mediated sodium transport	(51)
	*SCNN1B*	NG_011908.1	600760	miRNA-138-1-3p	Urine from healthy volunteers	βENaC-mediated sodium transport	(51)

Colon, smooth muscle cell, and Henle’s loop	*SLC12A2* (NKCC1)	NG_042286.1	600840	miRNA-26a-5p; miRNA16-5p; miRNA-181a-2-3p, miRNA-101-3p; miRNA-203a; miRNA-561-3p; miRNA-26b-5p; miRNA-15b-5p	Urine from healthy volunteers	NKCC1-mediated sodium, potassium, and chloride transport	(51)
				miRNA-15a-5p; miRNA-424-5p			
				miRNA-4524b-5p; miRNA-195-5p			
				miRNA-218-5p; miRNA-374b-3p			

Henle’s loop	*SLC12A1* (NKCC2)	NG_021301.1	600839	miRNA-16-5p; miRNA-561-3p; miRNA-3662; miRNA-335-3p; miRNA-15b-5p; miRNA-15a-5p; miRNA-424-5p; miRNA-195-5p; miRNA-548k	Urine from healthy volunteers	NKCC2-mediated sodium, potassium, and chloride transport	(51)

Proximal tubule cells	*AQP1*	NG_007475.2	107776	miRNA-128	Urine from healthy volunteers	Water balance	(51)

CDT cells	*AQP2*	NG_008913.1	107777	miRNA-4747-5p	Urine from healthy volunteers	Water balance	(51)

CDT cells	*NR3C2*	NG_013350.1	600983	miRNA-28-3p; miRNA-320-a; miRNA-205-5p; miRNA-431-5p; miRNA-421; miRNA-135a-5p; miRNA-409-3p; miRNA-186-5p; miRNA-211-5p; miRNA-129-5p; miRNA-873-3p; miRNA-204-5p	Urine from healthy volunteers	MR activation	(51)

Ubiquitous	*ICAM1*			miRNA-17	Rat urine from hypertension models (Ang II and l-NAME)	Vascular inflammation	(115)
	*LCoR*			miRNA-615-5p	Human urinary exosomes from salt-sensitive or inverse salt sensitivity patients	Upregulation of PPARγ	(49)
	*EGFR*			miRNA-221, miRNA-222	Human urinary exosomes from salt-sensitive or inverse salt sensitivity patients	EGFR pathway	(49)
	*PIK3R1, PTEN*			miRNA-29a-3p	Human urinary exosomes from salt-sensitive or inverse salt sensitivity patients	Blockade of the TGF-β PI3k–Akt pathway	(49)
	*AML1/ETO*			miRNA-193a-5p	Human urinary exosomes from salt-sensitive or inverse salt sensitivity patients	PTEN/PI3K signaling pathway	(49)

## Intrarenal Communication Mediated by Exosomes

Exosomes are proposed to play a key role in the inter- and intra-cellular communication of renal epithelial tissues among the different nephron segments. The available literature shows scarce and indirect evidence of MR activity associated to intrarenal communication mediated by exosomes. However, a study by Jella et al. (50) showed that apical and basolateral exosomes secreted from a proximal tubule cell line (LLC-PK1) carrying active GAPDH that was taken up by cortical collecting duct cells (mpkCCD), which decreased its ENaC activity. This effect was mimicked in Xenopus 2F3 (distal tubule cells) and cortical collecting duct cells from SV129 wild-type mice in a GAPDH-dependent manner (50), providing information on how exosomes released on the proximal portion of the nephron can influence the activity of sodium channels in distal portions of the nephron (see Figure 1).

Another example of exosomal transferring, come from mCCDC11 cells (from cortical collecting duct epithelia) stimulated with synthetic vasopressin analogs, which release exosomes loaded with AQP2 at levels that correlate with the AQP2 expression of the cell of origin. These exosomes are capable of transferring functional amounts of AQP2 to cells that do not express it, inducing an increase in cellular water flow (110). This suggests that exosomal content is physiologically regulated by vasopressin and other hormones, through the loading of exosomes with functional proteins capable of regulating water homeostasis. On the other hand, fenoldopam and Ang II stimulate exosome release from human renal proximal tubule cells, which can then be taken up by human distal and collecting tubule cells, where the exosomes accumulate into multivesicular bodies and modulate the activity of reactive oxygen species downstream (73).

Finally, normal human urinary exosomes isolated and sequenced by RNA sequencing revealed that miR-10b-5p, miR10a-5p, miR30a-5p, miR26a-5p, and miR-30d-5p were the most abundant urinary miRNAs (51), confirming some miRNAs previously reported by Cheng et al. (71). Afterwards, human collecting duct cells and proximal tubular cells (HKC-8) were exposed to these isolated urinary exosomes. They were internalized and reduced the protein levels of ROMK, SGK1 and WNK1 in human collecting duct cells, and decreased the mRNA levels of the coupled neutral amino acid transporter 2 *(SLC38A2)* and its encoded protein SNAT2 in HKC-8 cells (51). These studies showed a potential functionality of urinary exosomes through miRNAs, suggesting that they carry specific miRNA families that target specific renal functions.

## Conclusion

There is a growing evidence indicating that exosomes play a role in cardiovascular and renal physiology, where mineralocorticoid AHT could benefit from the discovery of effective biomarkers. Exosomes possess a variety of biological information, and urinary exosomes mainly carry RNA and proteins could mirror biological events in the kidneys, which can be a useful a tool for identifying and studying metabolic changes in renal physiological and pathophysiological conditions. This review shows current evidence about urinary exosomes carrying mRNA, miRNAs, and specific sodium channels (ENaC, NCC, NKCC2), which could reflect their abundance in renal tissue and be related to metabolic pathways associated with mineralocorticoid AHT. Therefore, the information carried by exosomes could be beneficial for diagnosing different subtypes of AHT and enabling more appropriate treatment and further improving the quality of life for patients. Although progress in recent years has been made to elucidate the role of exosomes, many questions regarding their specific functions of urinary exosomes along the nephron and their response to different stimulus and pathological conditions still need more comprehensive answers. Further studies are needed to determinate the potential benefits of exosomes in mineralocorticoid AHT.

## Author Contributions

EB and CC drafted the manuscript and prepared figures and tables, helped with writing the manuscript and designing the figure and table, critically reviewed and revised the manuscript, and read and approved the final version of the manuscript.

## Conflict of Interest Statement

The authors declare that the research was conducted in the absence of any commercial or financial relationships that could be construed as a potential conflict of interest.
